# Antioxidants of Edible Mushrooms

**DOI:** 10.3390/molecules201019489

**Published:** 2015-10-27

**Authors:** Maja Kozarski, Anita Klaus, Dragica Jakovljevic, Nina Todorovic, Jovana Vunduk, Predrag Petrović, Miomir Niksic, Miroslav M. Vrvic, Leo van Griensven

**Affiliations:** 1 Department for Chemistry and Biochemistry, Faculty of Agriculture, University of Belgrade, Nemanjina 6, Belgrade 11080, Serbia; E-Mail: maja@agrif.bg.ac.rs; 2 Department for Industrial Microbiology, Faculty of Agriculture, University of Belgrade, Nemanjina 6, Belgrade 11080, Serbia; E-Mails: aklaus@agrif.bg.ac.rs (A.K.); vampum00@yahoo.com (J.V.); mniksic@agrif.bg.ac.rs (M.N.); 3 Institute of Chemistry, Technology and Metallurgy, University of Belgrade, Njegoseva 12, Belgrade 11001, Serbia; E-Mails: djakovlj@chem.bg.ac.rs (D.J.); ninat@chem.bg.ac.rs (N.T.); mmvchem@sezampro.rs (M.M.V.); 4 Institute of Chemical Engineering, Faculty of Technology and Metallurgy, University of Belgrade, Karnegijeva 4, Belgrade 11060, Serbia; E-Mail: ppetrovic@tmf.bg.ac.rs; 5 Faculty of Chemistry, University of Belgrade, Studentski trg 12–16, Belgrade 11000, Serbia; 6 Plant Research International, Wageningen University and Research Centre, Droevendaalsesteeg 1, Wageningen 6700 AA, The Netherlands

**Keywords:** antioxidants, edible mushrooms, health, life quality, longevity, oxidative stress, reactive oxygen species

## Abstract

Oxidative stress caused by an imbalanced metabolism and an excess of reactive oxygen species (ROS) lead to a range of health disorders in humans. Our endogenous antioxidant defense mechanisms and our dietary intake of antioxidants potentially regulate our oxidative homeostasis. Numerous synthetic antioxidants can effectively improve defense mechanisms, but because of their adverse toxic effects under certain conditions, preference is given to natural compounds. Consequently, the requirements for natural, alternative sources of antioxidant foods identified in edible mushrooms, as well as the mechanistic action involved in their antioxidant properties, have increased rapidly. Chemical composition and antioxidant potential of mushrooms have been intensively studied. Edible mushrooms might be used directly in enhancement of antioxidant defenses through dietary supplementation to reduce the level of oxidative stress. Wild or cultivated, they have been related to significant antioxidant properties due to their bioactive compounds, such as polyphenols, polysaccharides, vitamins, carotenoids and minerals. Antioxidant and health benefits, observed in edible mushrooms, seem an additional reason for their traditional use as a popular delicacy food. This review discusses the consumption of edible mushrooms as a powerful instrument in maintaining health, longevity and life quality.

## 1. Introduction

Inadequate nutrition due to modern lifestyle and the increase of average longevity are the two key reasons for the growing incidence of disease all over the world. Oxidative stress caused by an imbalanced metabolism and an excess of reactive oxygen species (ROS) end into a range of disorders *i.e*., metabolic disease, heart disease, severe neural disorders such as Alzheimer’s and Parkinson’s, premature aging and some cancers. ROS are not only generated internally, in the organism, but also through various external sources like ultraviolet light, ionizing radiation, chemotherapeutics, inflammatory cytokines, and environmental toxins. Inhaling toxic chemicals from the environment has become unavoidable in modern civilization.

Apart of the endogenous antioxidant defense mechanisms of an organism, its dietary intake is another very important source of antioxidants and may contribute to oxidative homeostasis. Antioxidant supplements or antioxidant-containing foods may be used to help the organism to reduce oxidative damage as well to protect food quality by preventing oxidative deterioration. Just as in food production and packing, antioxidants are extensively used in health care, anti-aging and cosmetics. The growing preference for healthy food, cosmetics, and health and wellness products is influencing the growth of the antioxidants market. In addition, increasing demand for nutritional products and cosmetics obtained from natural sources is also driving the natural antioxidants market [1]. Population growth and the increasing healthcare spending levels have led to a consistent increase in the demand for antioxidant products. The global market for antioxidants is growing fast and is expected to more than double from $103.6 million in 2011 to reach $246.1 million in 2018 [2,3].

The antioxidants market by product type is segmented into natural antioxidants and synthetic antioxidants. Natural antioxidants are categorized into plant and fungal extracts, spices (rosemary, thyme, marjoram, oregano, sage, basil, pepper, clove, cinnamon, and nutmeg), flavonoids, ubiquinol (fully reduced form of coenzyme Q_10_), glutathione, zink (Zn), selenium (Se), vitamin A (including carotenoids), vitamin C and vitamin E (including tocopherols and tocotrienols) [4].

Synthetic phenolic antioxidants include butylated hydroxyanisole (BHA), butylated hydroxytoluene (BHT) and others e.g., propyl gallate, *tert*-butylhydroquinone (TBHQ), ethoxyquin (EQ), that all effectively inhibit oxidation [5]. However, some synthetic antioxidants may cause adverse toxic effects under certain conditions [6,7]. BHA, which is very often used as an additive in food industry, can have negative effects on the regulation of the activity of mitogen-activated protein kinase (MAPK) depending on the dosage [7,8]. Several synthetic antioxidants are authorized for use as feed additives in the European Union [9].

In recent years, the restriction on the use of synthetic antioxidants, such as BHA and BHT, has caused a rapidly increased interest towards natural antioxidant substances [6,7]. Requirements for natural alternative sources of antioxidant foods and ingredients derive primarily from consumers.

In recent years edible mushrooms have attracted attention as a commercial source of antioxidants [6,7,10]. They might be used directly in enhancement of antioxidant defenses through dietary supplementation to reduce the level of oxidative stress. There is a wealth of evidence to support the effectiveness of such a strategy *in vitro*.

Edible mushrooms include many fungal species that are either harvested wild or cultivated. Cultivated mushrooms as well as the more common wild mushrooms are often available in markets; porcini (*Boletus edulis*) or other ectomycorhyzal mushrooms may be collected on a smaller scale by private gatherers [11,12].

The true nutritive value of mushrooms has rapidly become known and recognized not only by mushroom researchers and farmers but also by the general consumers [13]. In addition to their good flavor, mushrooms possess favorable chemical composition with high amounts of functional proteins, low total fat level, and the high proportion of polyunsaturated fatty acids (PUFA), making them well suited for low calorie diets. Edible mushrooms provide a nutritionally significant content of vitamins (B_1_, B_2_, B_12_, C, D, and E) [14,15,16]. Moreover, mushrooms have a low glycemic index, and high mannitol, which is especially beneficial for diabetics. Mushrooms have very low sodium (Na) concentration, which is beneficial for hypertensive patients and a high content of potassium (K) and phosphorus (P), which is an important orthomolecular aspect [13]. In Asia mushrooms are used as important source of home remedies against various diseases elicited by oxidative stress [10].

There is no easy distinction between edible and medicinal mushrooms because many of the common edible species have therapeutic properties [12,17]. Besides antioxidant properties, mushrooms have received considerable attention for their biological activities, such as antitumor, antiviral, anticomplementary, anticoagulant, antidiabetic, hypolipidemic, hepatoprotective, immunostimulant and immunological activities, which made them suited for use in food, cosmetics, biomedicine, agriculture, environmental protection and wastewater management [7,18,19].

**Figure 1:**
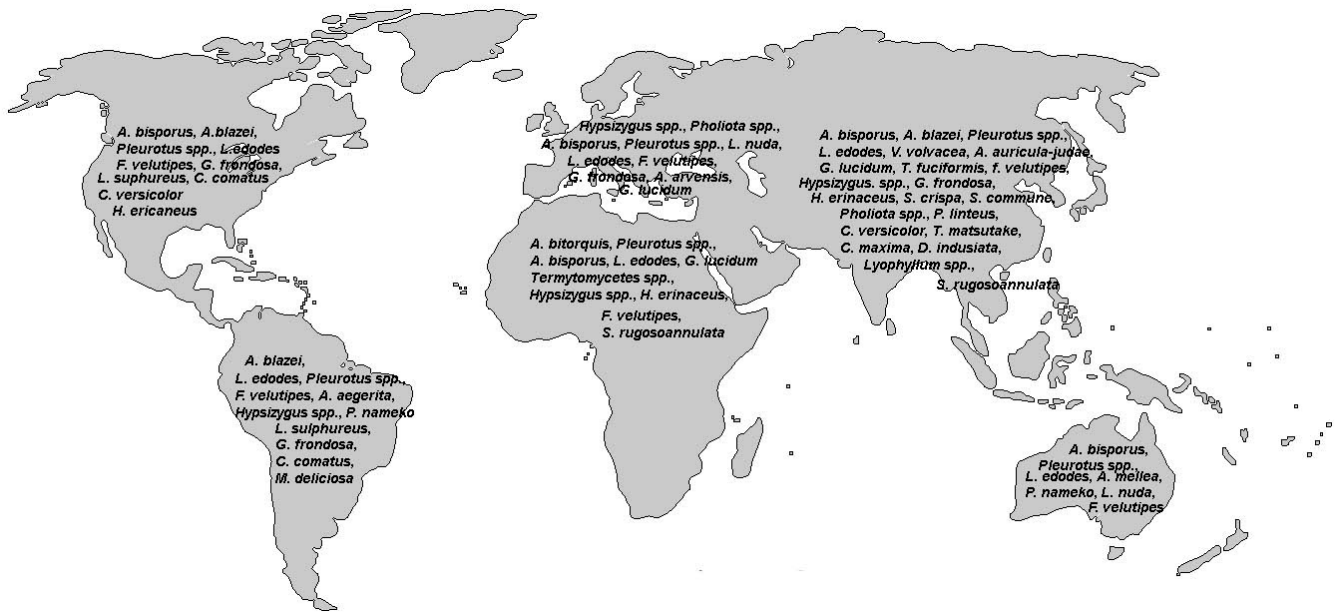
Map of edible mushroom species that are commonly grown commercially all over the world.

To date, a numerous edible wild mushroom species, growing in various ecological conditions, are known [20]. The common mushroom species produced in suitable ecological conditions are: *Agaricus* spp., *Lentinula edodes* (shiitake), *Pleurotus* spp. (oyster), *Volvariella volvacea* (straw), *Hericium erinaceus* (Lion’s head or pom pom), *Auricularia auricula-judae* (ear), *Grifola frondosa* (maitake), *Ganoderma lucidum* (lingzhi), *Flammulina velutipes*, *Tremella fuciformis*, *Pholiota nameko*, *Lepista nuda* (blewit) and *Coprinus comatus* (shaggy mane). Those of highest economic value are usually produced under artificial conditions, *i.e*., on a well defined substrate and under full climatization. These are mostly *Agaricus bisporus* (button mushroom), *Lentinula edodes*, *Pleurotus *spp., and *Flammulina velutipes* [20]. Mushrooms production is continuously increasing, with China being the biggest producer around the world [12] (Figure 1).

The Netherlands can be distinguished as the country at the forefront of European mushroom cultivation. Over the past 40 years a unique industry was established, resolute research carried on and exemplary education in mushroom growing was organized. The most important European country for the import of fresh and canned mushrooms is Germany. Mushroom production in Poland has increased intensely over the last 20 years and is now the largest in Europe [21].

In this work we review the antioxidant compounds identified in edible mushrooms, as well as the mechanistic aspects behind their antioxidant properties. The present review supplies a critical overview and is meant to promote further research and development of mushrooms.

## 2. ROS and Antioxidants in Cell Metabolism and Their Consequences in Human Cells and Health

### 2.1. Introduction to ROS

In the mid-1950s, Harman published a “Free-Radical Theory of Ageing”, speculating that endogenous oxygen radicals were generated in cells and resulted in a pattern of cumulative damage. When the supply of antioxidants was insufficient, Harman speculated, the resulting cell damage triggers a cascade of events that leads to disease development and death [14,22].

Since that hypothesis, our knowledge on involvement of free radicals and antioxidants in living processes has grown enormously. The field of free radicals or more common reactive species (RS) research in biological systems has become one of the most dynamic.

**Figure 2:**
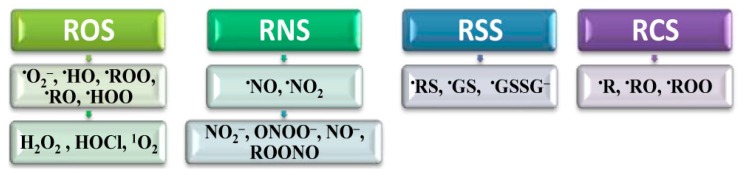
Classification of reactive species (RS) in living systems. Depending on the active center (*ac*) they are classified as: reactive oxygen species (ROS), *ac*-oxygen; reactive nitrogen species (RNS), *ac*-nitrogen; reactive carbon species (RCS), *ac*-carbon and reactive sulfur species (RSS), *ac*-sulfur.

Homeostasis is strongly influenced by many RS [23], such as ROS, reactive nitrogen (RNS), reactive carbon (RCS) and reactive sulfur species (RSS) (Figure 2). There are also many other RS consisting of halogens and related compounds [23].

ROS represent the most important class of reactive species generated in living systems [6,23]. In eukaryotic cells over 90% of ROS are produced by mitochondria via escape of electrons from the mitochondrial electron transport system (ETS), mainly from coenzyme Q to molecular oxygen (O_2_), resulting in the generation of superoxide anion radical (^•^O_2_^−^), named “primary” ROS. Further, ^•^O_2_^−^ spontaneously or enzymatically, can be dismutated into hydrogen peroxide (H_2_O_2_) and O_2_. H_2_O_2_ is not a free radical, but is chemically more active than O_2_ due to which it is included in the ROS group. It possesses the ability to form the more damaging ^•^OH, through a combination of the Fenton and Haber-Weiss reactions [23,24]. Finally, **^·^**OH interacts with one more electron and proton resulting in the formation of a water molecule (H_2_O). In biological systems, this reaction is mainly realized through abstraction of a hydrogen atom which originates from different compounds such as proteins and lipids, resulting frequently in initiation of radical chain processes. Besides of mitochondrial ETS, minor ROS are generated by ETS located in/at endoplasmic reticulum (ER), plasmatic, and nuclear membranes [23].

**Figure 3:**
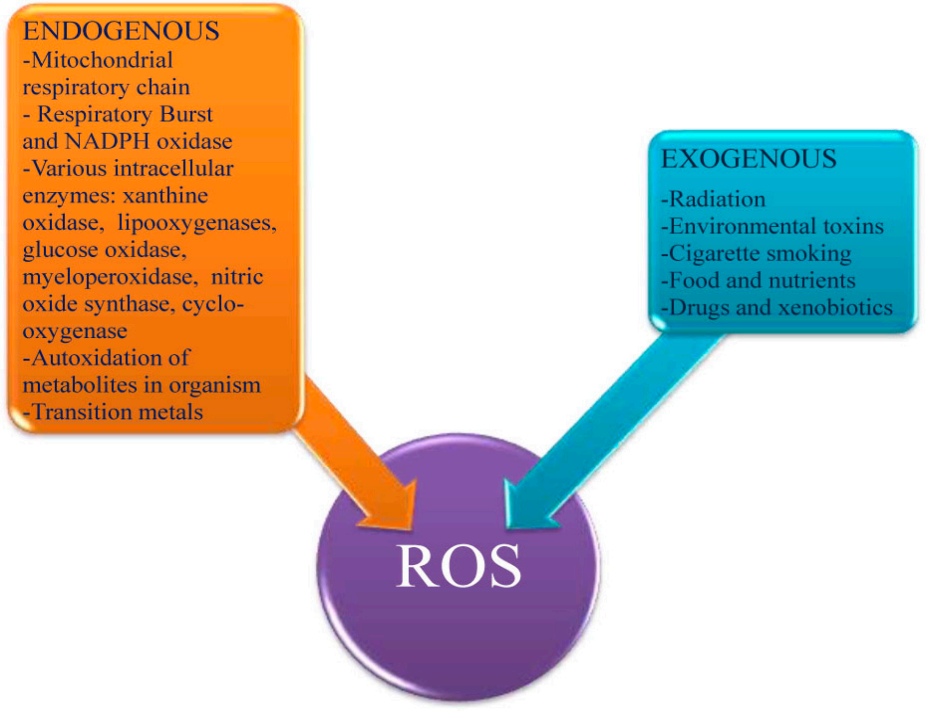
Endogenous and exogenous factors inducing ROS generation.

Diverse oxidases are also rather powerful ROS producers [23,25] (Figure 3). The physiological role of xanthine oxidase (XO) in total ROS balance, has received substantial attention. It is believed that under hypoxic conditions this enzyme may be the main ROS producer [26]. Additionally, autoxidation in the organism of different small molecules, such as adrenalin and norepinephrine, is also coupled with ROS production [27]. Even growth factors generate high levels of ROS that can perturb the normal redox balance and shift cells into a state of oxidative stress [14].

There are multiple external factors that induce ROS generation (Figure 3). Environmental pollution, radiation, cigarette smoking, certain foods, and drugs are the major exogenous sources of ROS. Many xenobiotics, especially different homo- and heterocyclic compounds, are clearly related as ROS generators in autooxidation reactions [6,23]. Inadequate nutrition, due to modern lifestyle, may also indirectly result in oxidative stress which impairs the cellular defense mechanisms.

### 2.2. Positive Effects of ROS in Homeostasis

ROS are a double-edge sword [28]. Despite their harmful effects the beneficial physiological cellular use of ROS is now being demonstrated in different fields [29]. Low physiological levels of ROS function as secondary messengers in intracellular signaling and are required for normal cell functions (Table 1). It is well documented that low levels of ROS can modulate cell proliferation, apoptosis, and gene expression through activation of transcription factors, like Nuclear Factor Kappa B (NF-κB) and hypoxia-inducible-factor-1α (HIF) [28,30,31,32,33,34].

**Table 1:** ROS signaling is integrated into many cellular pathways [28,29].

No.	Cellular Pathways
1	proliferation and survival pathways through mitogen-activated protein kinase (MAPK), phosphoinositide 3-kinase (PI3), phosphatase and tensin homolog (PTEN), and protein tyrosine phosphatases
2	ROS homeostasis and antioxidant gene regulation through redox effector factor-1 (Ref-1), NF-E2-related factor (Nrf-2), thioredoxin
3	Aging through p66Shc, a member of the Src homologous-collagen homologue (ShcA) adaptor protein family
4	DNA damage response through *ataxia-telangiectasia mutated kinase* (ATM); this may lead to inhibition of the mammalian target of the rapamycin complex 1 (mTORC1) resulting in suppression of protein synthesis and activation of autophagy
5	Iron homeostasis through iron-regulatory proteins (IRP) and iron-responsive elements (IRE)

A moderate increase of ROS may protect from infections caused by a broad range of microorganisms [35]. The production of ^•^O_2_^−^ and H_2_O_2_ by activated phagocytes is the classic example of the deliberate metabolic generation of ROS for useful purposes [28,36]. Moderate amounts of mitochondrial ^•^O_2_^−^ and H_2_O_2_ have important roles in a range of cellular signaling processes and can activate signaling pathways that promote cell survival and disease resistance due to hormesis [28,37]. Generation of ^•^O_2_^−^, HOCl, and H_2_O_2_ by phagocytes is important for defense against various bacterial and fungal strains [38]. For example individuals with chronic granulomatous disease who have deficiencies in generating ROS, are highly susceptible to infection by a broad range of microbes including *Salmonella enterica*, *Staphylococcus aureus*, *Serratia marcescens*, and *Aspergillus* spp. [39,40]. **^·^**O_2_^−^ is produced also by several cell types other than phagocytes, including lymphocytes and fibroblasts [28].

Detoxification reactions, ensured by the cytochrome P450 family, are dependent on the integrity of the microsomal ROS generating system. Nicotinamide adenosine dinucleotide phosphate (NADPH) supplies electrons, required for the reduction of O_2_ and the formation of ROS by microsomal monooxygenases, which have cytochrome P450 as a central link. They oxidize hydrophobic toxic substances, steroids and drugs, transforming them into hydrophilic compounds, which are removed from the body [41,42].

Further, a moderate increase of ROS is involved in apoptosis which eliminates cancerous and other life-threatening cells [35]. Apoptosis sometimes called “a guardian angel” or “cell policeman” [42], is carried out by a multistage chain of reactions, arises in cancer cells, in which ROS act as triggers and essential mediators [42].

Mitochondria play a critical role in apoptosis [43]. Apoptotic signals promote accumulation of the p53 protein that triggers the release of ROS, cytochrome C and a few other regulators from mitochondria [42]. ROS can act as signaling intermediates for cytokines, including interleukin 1 (IL-1) and the tumor necrosis factor (TNF) family [28,44,45,46]. Members of the TNF cytokine family, such as TNFα and Fas ligand (FasL), play important roles in inflammation and immunity [47,48]. Although TNF-related apoptosis-inducing ligand (TRAIL) is a member of this family, this molecule can induce (most, but not all) cancer cell death by its binding to the death receptors (DR), while causing almost no cytotoxicity to normal cells [47,49,50]. Normal cells are reported to show TRAIL-resistance with their preferential expression of decoy receptors (DcRs), which inhibit apoptotic signaling [51].

TRAIL-induced activation is a cascade of proteolytic enzymes, called caspases, which digest a number of cancer cell proteins and promote a caspase-activated deoxyribonuclease. Cleavage of the critical proteins and deoxyribonucleic acid (DNA) results in apoptotic cell death [42]. Also some reports suggest that TRAIL can induce necroptosis in cancer cells via ROS signaling [47,52,53,54].

In modern medicine generation of ROS by prooxidant compounds increasingly attracts attention as a therapeutic strategy for cancer [35]. For example, the compound 4-benzyl-2-methyl-1,2,4-thia-diazolidine-3,5-dione (TDZD-8) can induce depletion of thiols and rapid accumulation of ROS and selectively kill leukemic cells that express stem-cell markers, with minimal toxicity to normal hematopoietic stem cells [55]. Because tumor stem cells are thought to be the subpopulation of cells that are highly resistant to chemotherapy and play a critical role in disease relapse after treatment, the potency of the prooxidative compound in removing these cells underscores a key role of the redox system in regulating survival of stem cells and highlights the promising therapeutic potential of using a redox-based strategy in cancer treatment [35].

A transient increase of levels of oxidative stress by physical exercise reflects a potentially health promoting process at least in regards to prevention of insulin resistance and type 2 diabetes mellitus [56]. Physical training has been shown to improve glucose metabolism [57] by inducing molecular regulators of insulin sensitivity and endogenous antioxidant defense [56]. Exercise, as well as weight loss, has been linked to activation of mitochondrial metabolism, and reduced mitochondrial metabolism has been functionally connected with type 2 diabetes [58]. Muscle tissue is also known to generate ROS, especially during contraction and physical exercise [59].

### 2.3. Elimination of ROS in Living Systems

**Figure 4:**
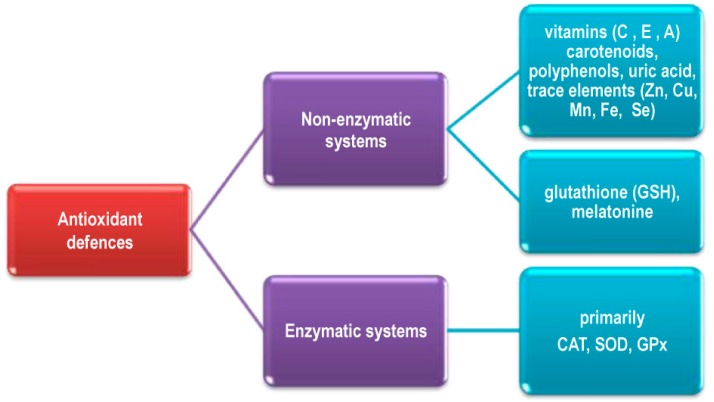
Major ROS defense mechanisms in the organism.

Living organisms possess a multilevel and complicated antioxidant system operating either to eliminate ROS, or to minimize their negative effects. Major ROS defense mechanisms include enzymatic and nonenzymatic systems (Figure 4). The ROS which are not neutralized can target biological molecules such as DNA, lipids and proteins, which can result in cell death or dysfunction and accelerated ageing and age-related diseases [14,23].

There are many different endogenous enzymatic antioxidant defense systems in the organism, either in intracellular or extracellular medium. They primarily include superoxide dismutase (SOD), catalase (CAT), glutathione peroxidases (GPx), and glutathione redutase (Gred) (Figure 4). Red blood cells are particularly sensitive to oxidative environments throughout the body and, as a consequence of their iron (Fe) content, are capable of producing their own ROS. However, the presence of antioxidant enzymes such as SOD and CAT, and methemoglobin reductase which catalyzes the reduction of methemoglobin to hemoglobin, minimized these processes [24,60]. The red blood cells and hepatocytes possess the highest level of CAT activity in the human body [60,61].

Nonenzymatic ROS defense mechanisms include low molecular mass antioxidants such as vitamins C and E, carotenoids including vitamin A, polyphenols, uric acid, (Figure 4) and large molecules *i.e*., albumin, ceruloplasmin, transferrin, ferritin [62]. In addition, Zn, copper (Cu), manganese (Mn), Fe, and Se are key components of enzymes with antioxidant functions and are designated as antioxidant micronutrients. Most of them are available to the human organism as food or supplement components [23,25,62,63,64].

Very important nonenzymatic antioxidants are glutathione (GSH), synthesized by most living organisms, and melatonin. As melatonin can directly cross the mitochondrial membranes, it plays a very significant role in the protecting mitochondria from oxidative damage. Probably due to antioxidant activity melatonin can improve glucose metabolism via correction of insulin production protecting pancreatic β-cells against ROS induced damage [65]. Other hormones. *i.e*., estrogen and angiotensin, also express antioxidant activity [63,66,67].

Alterations of the balance between ROS production and the capacity to rapidly detoxify reactive intermediates lead to oxidative stress. It has been implicated in a wide variety of states, processes and disease, e.g., aging, ischemia-reperfusion (I/R) injury, muscle damage, hypertension, atherosclerosis, diabetes, renal diseases, liver diseases, neurological diseases including Parkinson’s disease, Alzheimer’s disease and other forms of dementia, as well as diverse cancers. Oxidative stress also contributes to various gastrointestinal (GI) diseases including gastroduodenal ulcers, inflammatory bowel disease, and GI malignancies such as gastric and colorectal cancer [23,25,68].

### 2.4. Regulation of Antioxidant Systems

Under oxidative stress, an organism develops responses to prevent or neutralize negative ROS effects. These responses are mainly based on up-regulation of antioxidant and related enzymes. Mechanisms include ROS sensing, transduction of signals through specific pathways and up-regulation of target genes to enhance level of their products [23].

**Figure 5:**
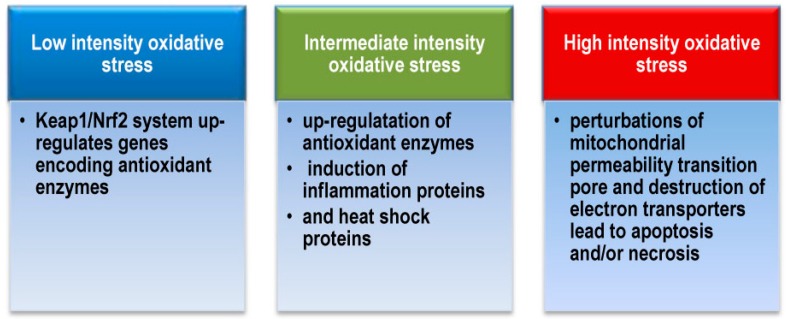
Human and animal organism system of adaptive response to ROS exposure.

The human organism possesses a complicated system of adaptive responses to ROS exposure. Usually, it has several components (Figure 5). Under low intensity oxidative stress, the Kelch-like ECH-associated protein 1/NF-E2-related factor 2 (Keap1/Nrf2) system up-regulates genes encoding antioxidant enzymes. It is known to be activated by minute amounts of ROS. Intermediate intensity oxidative stress up-regulates antioxidant enzymes and induces inflammation proteins and heat shock proteins via NF-κB, activator protein-1 (AP1), MAPKs and heat shock factor (HSF). Under high intensity oxidative stress perturbations of mitochondrial permeability transition pore, activation of apoptosis cascade, destruction of electron transporters take place, which may culminate in apoptosis and/or necrosis [23,69].

Up-regulation of antioxidant systems increases their capability to eliminate ROS creating in this way autoregulated negative feedback control loop.

## 3. Antioxidant Compounds in Mushrooms

A whole range of edible mushrooms were reported to possess antioxidant activity (Table 2). It is generally accepted that extracts of fungi contain many components, each of which has its own specific biological effects [70,71]. Antioxidant compounds found in fruit bodies, mycelium and broth confirmed to be phenolics, flavonoids, glycosides, polysaccharides, tocopherols, ergothioneine, carotenoids, and ascorbic acid [16,17,72,73,74,75,76,77,78,79,80,81,82,83,84,85,86,87,88,89,90,91,92,93,94,95,96,97,98,99,100,101,102,103,104,105,106,107,108,109,110,111,112,113,114,115,116,117,118,119,120,121,122,123,124,125,126,127,128,129,130,131,132,133,134,135,136,137,138,139,140,141,142,143,144,145,146,147,148].

**Table 2:** Some studies of antioxidative properties of wild and cultivated mushrooms.

Mushroom Species	References
*Agaricus bisporus, Agaricus brasiliensis* (=*Agaricus blazei ss. Heinem.*), *Agrocybe aegerita*, *Auricularia auricular*, *Auricularia cornea*, *Auricularia polytricha*, *Auricularia mesenterica*, *Auricularia fuscosuccinea*, *Agrocybe cylindracea*, *Amanita rubescens*, *Agaricus arvensis*, *Armillariella mellea*, *Agaricus silvicola*, *Agaricus silvaticus*, *Agaricus romagnesii*, *Antrodia camphorate*	[75,77,81,82,83,90,91,93,97,103,104,112,115,117,121,123,125,129,130,131,132,133,135,139,140,142,144]
*Boletus edulis*, *Boletus badius*	[91,123,144]
*Cantharellus lutescens*, *Cantharellus clavatus*, *Cantharellus cibarius*, *Cordyceps sinensis*, *Calvatia gigantea*, *Cerrena unicolor*, *Coprinus comatus*	[16,91,93,97,104,115,134,140,142,144,147]
*Dictophora indusiata*	[114]
*Flammulina velutipes* (white), *Flammulina velutipes* (yellow)	[95,103,105,136,137,138]
*Inonotus obliquus*	[78,79,80]
*Ganoderma lucidum*, *Ganoderma tsugae*, *Grifola frondosa*, *Ganoderma applanatum*, *Geastrum arenarius*, *Geastrum saccatum*, *Ganoderma atrum*	[74,75,76,86,87,88,89,91,93,94,96,98,99,107,109,110,111,114,118,119,127,142,145,146]
*Hericium erinaceus*, *Hericium coralloides*, *Hydnum repandum*, *Hygrophorus agathosmus*, *Hypsizigus marmoreus*, *Hypholoma fasciculare*, *Helvella crispa*	[91,97,103,113,114,115,123,140,147]
*Lepista nuda*, *Lentinus edodes*, *Lactarius sanguifluus*, *Lentinus squarrosulus*, *Lactarius deliciosus*, *Lentius sajor-caju*, *Leucopaxillus giganteus*, *Lactarius piperatus*, *Laetiporus sulphureus*, *Lycoperdon molle*, *Lycoperdon perlatum*, *Lactarius piperatus*	[73,74,81,84,91,95,97,99,103,104,106,115,120,122,125,126,128,133,140,144,145,147]
*Morchella esculenta*, *Morchella conica*, *Macrolepiota procera*, *Morchella angusticeps*, *Macrolepiota procera*	[85,91,118,140]
*Pleurotus ostreatus*, *Pleurotus eryngii*, *Pleurotus citrinopileatus*, *Pleurotus djamor*, *Pleurotus sajor-caju*, *Pleurotus cystidiosus*, *Pleurotus australis*, *Pleurotus tuber-regium*, *Phellinus linteus*, *Phellinus rimosus*, *Phellinus merrillii*, *Polyporus squamosus*, *Picoa juniperi*, *Pleurotus florida*, *Pleurotus pulmonarius*, *Paecilomyces japonica*, *Piptoporus betulinus*	[50,75,81,88,91,92,93,95,97,102,103,104,108,115,116,124,133,141,142,144,148]
*Russula brevipes*, *Russula cyanoxantha*, *Russula delica*, *Ramaria botrytis*, *Russula vinosa*	[91,101,104,123,140]
*Sparassis crispa*, *Suillus bellini*, *Suillus luteus*, *Suillus granulatus*, *Sarcodon imbricatus*, *Schizophyllum commune*	[72,91,123,125,140,147]
*Tricholoma acerbum*, *Tricholoma equestre*, *Tricholoma giganteum*, *Tricholomopsis rutilans*, *Termitomyces microcarpus*, *Termitomyces schimperi*, *Termitomyces mummiformis*, *Termitomyces tylerance*, *Termitomyces heimii*, *Termitomyces albuminosus*, *Termitomyces robustus*, *Terfezia claveryi*, *Tremella fuciformis*, *Trametes (Coriolus) versicolor*, *Trametes orientalis*	[74,90,91,93,99,100,114,115,118,123,133,140,145,147]
*Verpa conica*, *Volvariella volvacea*	[103,104,106,120]

The various methods used in order to measure the antioxidative properties of mushroom compounds or extracts are appropriate for various levels of antioxidative activity, such as methods based on the transfer of electrons and hydrogen atoms, the ability to chelate ferrous (Fe^2+)^ and cupric (Cu^2+^) ions, the electron spin resonance (ESR) method, erythrocyte hemolysis, and the monitoring of the activity of SOD, CAT and GPx [16,17,72,73,74,75,76,77,78,79,80,81,82,83,84,85,86,87,88,89,90,91,92,93,94,95,96,97,98,99,100,101,102,103,104,105,106,107,108,109,110,111,112,113,114,115,116,117,118,119,120,121,122,123,124,125,126,127,128,129,130,131,132,133,134,135,136,137,138,139,140,141,142,145,146,147].

It has been established that mushroom antioxidants can demonstrate their protective properties at different stages of the oxidation process and by different mechanisms [4,6,7]. There are two main types of mushroom antioxidants, namely, primary (chain breaking, free radical scavengers) and secondary or preventive [4,6,7,16,72,73,74,75,76,77,78,79,80,81,82,83,84,85,86,87,88,89,90,91,92,93,94,95,96,97,98,99,100,101,102,103,104,105,106,107,108,109,110,111,112,113,114,115,116,117,118,119,120,121,122,123,124,125,126,127,128,129,130,131,132,133,134,135,136,137,138,139,140,141,142,145,146,147]. Secondary antioxidants are the consequence of deactivation of metals, inhibition or breakdown of lipid hydroperoxides, regeneration of primary antioxidants, singlet oxygen (^1^O_2_) quenching, *etc*. Some mushroom substances that exhibit antioxidant activity function as inducers and/or cell signals, leading to changes in gene expression, which result in the activation of enzymes that eliminate ROS [6,108,109,110,111,149].

Different analytical methods have been applied for its identification and quantification: high performance liquid chromatography (HPLC) and gas chromatography (GC) coupled to distinct detection devices, nuclear magnetic resonance (NMR), Fourier transform infrared (FT-IR), UV-VIS spectroscopy and various spectrophotometric assays [16,17,72,73,74,75,76,77,78,79,80,81,82,83,84,85,86,87,88,89,90,91,92,93,94,95,96,97,98,99,100,101,102,103,104,105,106,107,108,109,110,111,112,113,114,115,116,117,118,119,120,121,122,123,124,125,126,127,128,129,130,131,132,133,134,135,136,137,138,139,140,141,142,143,144,145,146,147,148].

### 3.1. Polyphenols Including Flavonoids

Polyphenols are the most abundant antioxidants in the diet [150]. Research on the effects of dietary polyphenols on human health has developed considerably in the past 20 years. One of the major difficulties of elucidating the health effects of polyphenols is the large number of phenolic compounds found in food [150,151], yielding differing biological activities [152].

These compounds may be classified into different groups as a function of the number of phenol rings and the structural elements which bind these rings to each other. Thus a distinction is made between the phenolic acids, flavonoids, stilbenes, and lignans. In addition to this diversity, most polyphenols are present in food in the form of esters, glycosides, or polymers. These substances cannot be absorbed in their native form and must be hydrolyzed by intestinal enzymes or by the colonic microflora before absorption. In the course of absorption, polyphenols are conjugated and this process mainly includes methylation, sulfation, and glucuronidation. Circulating polyphenols are conjugated derivatives that are extensively bound to albumin [153]. Nonconjugated metabolites are generally either absent from the blood or present in low concentrations [152]. Major differences in bioavailability of polyphenols are now well established facts and the influence of structural factors is better understood [152,153,154].

The main phenolic compounds found in mushrooms are phenolic acids [6]. Phenolic acids can be divided into two major groups, hydroxybenzoic acids and hydroxycinnamic acids, which are derived from the non-phenolic molecules benzoic and cinnamic acid, respectively [6,153,154].

Hydroxybenzoic acid derivatives commonly occur in the bound form and are typically a component of a complex structure like lignins and hydrolyzable tannins. They can also be found linked to sugars or organic acids. Hydroxycinnamic acid derivatives are mainly present in the bound form, linked to cell-wall structural components, such as cellulose, lignin, and proteins, as well as associated to organic acids, such as tartaric or quinic acids (*i.e.*, chlorogenic acids), through ester bonds [6,153].

The hydroxycinnamic acids are more common than the hydroxybenzoic acids and consist chiefly of *p*-coumaric, caffeic, ferulic, and sinapic acids. These acids are rarely found in the free form, except in processed food that has undergone freezing, sterilization, or fermentation. The bound forms are glycosylated derivatives or esters of quinic acid, shikimic acid, and tartaric acid [153,154].

The most common (prevalent) benzoic acid derivatives found in mushrooms are reported to be *p*-hydroxybenzoic, protocatechuic, gallic, gentisic, homogentisic, vanillic, 5-sulphosalicylic, syringic, veratric, vanillin [6]. The majority of identified cinnamic acid derivatives in mushrooms are: *p*-coumaric, *o*-coumaric, caffeic, ferulic, sinapic, 3-*o*-caffeoylquinic, 4-*o*-caffeoylquinic, 5-*o*-caffeoylquinic [6]. Besides, presence of ellagic and tannic acids is observed [6].

Natural polyphenolic compounds exert their antioxidant effect by quenching free radical species and/or promoting endogenous antioxidant capacity. Furthermore, some of them stimulate synthesis of endogenous antioxidant molecules in cells via activating the Nrf/ARE pathway [149,150]. Cells respond to polyphenols mainly through direct interactions with receptors or enzymes involved in signal transduction, which may result in modification of the redox status of the cell and may trigger a series of redox-dependent reactions [150,155,156,157].

Apart from the antioxidant capacity, most of these compounds appear to have a number of different molecular targets, impinging on several signaling pathways, and showing pleiotropic activity on cells. For instance, polyphenolics can modulate activity of NF-κB or Sirutin1 (SIRT1) exerting neuroprotective effects [154,158]. SIRT1 acts as a “rescue gene”, capable to repair damages caused by the action of free radicals and to prevent premature death of cells. The gene also affects the mitochondria to produce greater amounts of energy what is typical for the metabolism of younger cells. As a result, SIRT1 is believed to be a principal regulator of lifespan [158,159].

Besides antioxidant properties, polyphenols possess pro-oxidative capacity based upon the structure of the particular polyphenol and the cellular redox context that may include increased levels of oxidant scavenging proteins or decreased levels of oxidized proteins and lipids [159]. For example, epigallocatechin-3-gallate (EGCG) induces the activation of protein caspases-3 and c-Jun *N*-terminal kinases (JNKs), which belong to the group of MAPKs that have a role in the process of programmed cell death, *i.e*., apoptosis. These various effects are dependent on cell type, stress conditions, concentrations and the time of exposure of EGCG. It is possible that low concentrations of EGCG activate MAPK, leading to antioxidant responsive element (ARE)-mediated gene expression, whereas higher concentrations and sustained activation of MAPKs lead to apoptosis [7,160].

Polyphenols such as tannic acid, gallic acid, and catechin compounds are also known to interact with steroid receptors. This may lead to a change of the mitochondrial transmembrane potential and ultimately to a decrease or increase in ROS activation, depending on cell systems [142]. As antioxidants, polyphenols may improve cell survival; as prooxidants, they may induce apoptosis and prevent tumor growth, and combat against bacterial and viral infections by direct oxidative damage or by a variety of innate and adaptive mechanisms [150,161].

Recently, Lagunes and Trigos [162] showed that polyphenols, e.g., curcumin, resveratrol and quercetin, have pro-oxidant activity; they act as photosensitizers in the generation of ^1^O_2_. Resveratrol and curcumin have been reported as efficient quenchers of ^1^O_2_ [163,164]. On the other hand, they have also been reported as molecules capable of absorbing ultraviolet-visible (UV-Vis) light and able to transfer it to O_2_ to generate ^1^O_2_ [162,165]. Recent studies have shown that curcumin is able to inhibit melanoma cells proliferation, under UVA-Vis light, suggesting that it could be used as an adjunctive therapy in the treatment of cancerous diseases [166]. Resveratrol and curcumin are lipophilic molecules and mix very well between membrane lipids and lipoproteins; their levels in tissues may be higher than those detected in blood [167]. High consumption of dietary supplementation with resveratrol and curcumin could lead to a high bioavailability in tissues and when these are exposed to light, it would allow resveratrol and curcumin to act as photosensitizers, showing a pro-oxidant activity by generation of ^1^O_2_. ^1^O_2_ is a powerful oxidizing agent initiator of oxidation processes in biological systems [168], by which resveratrol and curcumin would favour a pro-oxidant effect against normal cells [162]. Analogous processes are likely to occur with lipophilic polyphenols of mushrooms, which requires attention should be given to possible harmful effects.

#### Flavonoids

Flavonoid research has intensified since the discovery of the “French paradox” [169,170]. This phenomenon was observed in the French population as a significantly lower rate of heart diseases despite high consumption of saturated fat and smoking habits and was related to moderate and regular consumption of red wine rich in flavonoid compounds [170].

Basic structure of flavonoid has a flavan nucleus consisting of two benzene rings (A and B) combined by an oxygen-containing pyran ring (C). The various classes of flavonoids differ in their level of oxidation of the C ring of the basic 4-oxoflavonoid (2-phenyl-benzo-γ-pyrone) nucleus. The common six subclasses of flavonoids are flavonols, flavones, isoflavones, flavanones, anthocyanidins, and flavanols [153,169,171]. Flavonoids are most frequently found in nature in the form of glycosylate or esterificate conjugates but can also occur as aglycones in food, especially as a result of the food processing. Flavonols are the most abundant flavonoids in foods [170,171].

In general, it can be assumed that only plants possess the biosynthetic ability to produce flavonoids, while animals and fungi are not capable of it [6]. However, the presence of flavonoids is reported in different edible mushrooms, e.g., myricetin, chrysin, catechin, hesperetin, naringenin, naringin, formometin, biochanin, pyrogallol, resveratrol, quercetin, rutin, kaempferol [6]. More recently, the analysis of the methanolic extract of *Cantharellus cibarius* showed that phenols were its major antioxidant components but followed by flavonoids, whose content was approximately 86% of the total phenol content [16].

Mechanisms of the antioxidant action of flavonoids can include direct scavenging of RS, chelating of trace metal ions involved in RS formation, inhibition of enzymes, e.g., XO and lipoxygenases (LOXs), involved in RS production, and regeneration of membrane-bound antioxidants such as α tocopherol [169]. It is generally considered that the primary mechanism of the radical scavenging activity of flavonoids is hydrogen atom donation. [170,171].

The capacity of flavonoids to inhibit ROS is governed by the presence and position of the multiple hydroxyl groups in their structure. A double bond and carbonyl function in the heterocycle or polymerization of the nuclear structure increases activity by affording a more stable flavonoid radical through conjugation and electron delocalization [170,171]. Mechanisms of flavonoid action, such as modulation of signalling pathways and gene expression, could also contribute to protective properties of flavonoids [153].

Further, flavonoids may act as pro-oxidants based upon on a different number of OH groups in the B ring and presence of transient metal ions [170]. The flavonoid phenoxyl radical could interact with O_2_, generating quinones and superoxide anion (O_2_^−^), rather than terminating the chain reaction. This reaction may take place in the presence of high levels of transient metal ions and may be responsible for the undesired prooxidant effect of flavonoids [170].

El Amrani *et al*. [172] reported oxidative DNA cleavage induced by binding of a Fe^3+^-flavonoid complex to DNA. Likewise, reacting with Cu^2+^ quercetin induces extensive DNA damage, but kaempferol and luteolin induce only slight DNA damage, even in the presence of Cu^2+^ [170]. These complexes may play an important role in their potency for biological action such as angiogenesis and immune-endothelial cell adhesion, which, respectively, are important processes in the development of cancer and atherosclerosis [173].

Also, it has been shown that quercetin could aggravate the severity of renal cell carcinoma through the tumorigenic action mechanism due to pro-oxidant effect [174]. It act as an antioxidant in low doses, or as pro-oxidant at higher doses [175].Concerning the absorption and bioavailability of quercetin, it has been demonstrated that in an aglycosylated form it becomes a lipophilic molecule and can be easily absorbed through epithelia in cells of the colon [162,176]. In a similar manner to resveratrol, quercetin would be able to be incorporated into cellular membranes, and under specific conditions, show a pro-oxidant activity by generation of ^1^O_2_ [162].

### 3.2. Polysaccharides

Polysaccharides, including polysaccharide-protein complexes, have been recognized as a class of major bioactive constituents of edible and medicinal mushrooms [70,71]. They act primarily as adaptogens and immunostimulators. The immunostimulatory effect of mushroom polysaccharides is prophylactic primarily and it belongs to non-invasive treatments, playing a role in the prevention of infectious diseases and of tumor metastases [7,70,71,177,178].

Another widely reported activity of mushroom polysaccharides is antioxidative [7,72,73,74,75,76,77,99,145]. Many mushroom polysaccharides have been reported to have significant antioxidant activities based on various *in vitro* and *in vivo* assays [7,179]. Their antioxidative activity is attributed to the ability of RS scavenging, their reduction property and ability to chelate Fe^2+^, lipid peroxidation inhibition, erythrocyte hemolysis and the increase of enzymes activities in eukaryotic as well as in prokaryotic cells as well as in taking part in antioxidative processes, such as SOD, CAT and GPx [7,108,109,110,111].

The extraction procedure and the method used for the purification of the obtained fractions of polysaccharides depend in many aspects on the type of a mushrooms and physical properties of polysaccharides. Usually these polysaccharides were isolated from the hot-water extract simply by ethanol precipitation without further purification [7,72,73,74,75,76,77,99]. Purification of the crude polysaccharides is usually accomplished by chromatographic methods such as size-exclusion (SEC) and ion-exchange chromatography (IEC) [99,178], as well as by enzymatic purification using cellulases, amylases and proteases [7,77]. Besides, new extraction procedures were reported, as ultrasonic-assisted extraction [145,180] and more recently, extraction procedure with a freeze-thawing process [181]. Klaus *et al*. [76] have managed to encapsulate polysaccharide extracts from *G. frondosa* in alginate gel beads to protect them from external influences and this could contribute to a more widespread use.

Some studies have found that the purified mushroom polysaccharides had lower antioxidant activities than the original crude extracts [99,146]. Polysaccharides in the fungal cell wall may be bound by covalent (ester) linkages with proteins via remains of tyrosine and/or with ferulic acid as a result of lignin degradation processes [7,74]. However, other studies reported higher antioxidant activity in the pure polysaccharide fraction, e.g., the *A. brasiliensis* polysaccharides obtained by pronase deproteinization, demonstrated a high antioxidative activity against ^•^OH and ^•^O_2_^−^ radicals measured by electron paramagnetic resonance (EPR) spin-trapping spectroscopy. These polysaccharides consisted mainly of (1→6)-β-d-glucans [77].

The major antioxidant effects of mushrooms are attributed to β-glycans. Except reported antioxidant properties, α-glycans are eukaryotic nutrient components and are easily degraded by mammalian enzymes. β-Glycans from various mushrooms can be taken up by the M cells of Peyer’s patches, and/or interact with dendritic cells (DCs) in the small intestine to activate systemic organism responses [182,183].

The ability of the polysaccharide molecules to scavenge free radicals may be conditioned by the presence of hydrogen from specific, certain monosaccharide units, and the type of their binding in side branches of the main chain [84,184]. The enhanced antioxidant activity of the polymers over the monomeric form may be due to the greater ease of abstraction of the anomeric hydrogen from one of the internal monosaccharide units rather than from the reducing end [184].

Recently, Kishk and Al-Sayed [185] reported that the ^•^OH scavenging mechanism of polysaccharides was perhaps similar to that of phenol compounds by hydrogen atom transfer (HAT) reactions. However, the HAT reaction is more likely to occur in the neutral polysaccharides, while the electron transfer (ET) mechanism is usually occur in the acidic polysaccharides.

Mushroom polysaccharides and glycoconjugates may be useful in creating new natural-based medications or dietary supplements and helpful in the prevention and treatment of oxidative stress-mediated disorders. ROS are produced within the GI tract. Despite the protective barrier provided by the mucosa, ingested materials and microbial pathogens can induce oxidative injury and GI inflammatory responses involving the epithelium and immune/inflammatory cells. High antioxidative capacities of polysaccharides from edible mushrooms can prevent lipid peroxidation [72,73,74,75,76,77] and the pathogenesis of various GI diseases including peptic ulcers [186], GI cancers [182], and inflammatory bowel disease which is in part due to oxidative stress [25].

Cytotoxic effects of polysaccharide extracts of higher fungi on normal cells have not been reported up-to-date [7,73,77]. Furthermore, it was confirmed that polysaccharide extract of *G. lucidum*, *in vitro*, stimulates proliferation of HTR-8/SVneo trophoblast cells which are essential for normal placentation, establishment of pregnancy and maintenance of fetal growth in humans [73,187].

The polysaccharide extracts proved to be heat stable and retained high antioxidant potential, despite all the treatments applied in their preparation [72,73,74,75,76,77].

### 3.3. Vitamins

#### 3.3.1. Vitamin C

Vitamin C, also known as l-ascorbic acid, is the primary antioxidant in plasma and cells [25]. It is an essential nutrient for a limited species of animals, including humans, and therefore must be ingested to avoid a potentially lethal condition [6,25,188]. Likewise, ascorbic acid is a normal skin constituent found at high levels in both the dermis and epidermis [189,190]. Ageing, however, causes a decline in vitamin C content in both layers. Excessive exposures to UV light or pollutants (e.g., cigarette smoke and ozone) may also lower its content, primarily in the epidermis [189,190].

Vitamin C was detected in various edible mushrooms [6,16]. It was quantified by HPLC or spectrophotometric assay, based on the reaction with 2,6-dichlorophenolindophenol [6,16]. Kozarski *et al*. [16], found 100 mg/100 g dry weight (DW) of ascorbic acid in the methanolic extract of the wild edible mushroom *Cantharellus cibarius*. It was higher than its content in some fruits and vegetables which are usually recommended as a good source of vitamin C [191]. Reported amount of ascorbic acid found in strawberries is 60 mg/100 g, citrus fruits 30–50 mg/100 g, while apples, pears and plums represent only a very modest source of ascorbic acid (3–5 mg/100 g) [191].

Grangeia *et al*. [192] found high concentrations of ascorbic acid in the methanolic extracts of different saprotrophic and mycorrhizal wild edible mushrooms (81.32–400.36 mg/100 g DW).

Ascorbic acid has been shown as an effective RS scavenger [188,191]. In studies with human plasma lipids it appeared that ascorbate was far more effective in inhibiting lipid peroxidation initiated by a lipid peroxyl radical (^•^LOO) initiator than other plasma components, such as protein thiols, urate, bilirubin, and vitamin E. In the aqueous phase, ascorbic acid can protect biomembranes against peroxidative damage by the efficiently trapping ^•^LOO before they can initiate lipid peroxidation [188,191]. It may also recycle vitamin E, the main lipid-soluble antioxidant. Ascorbic acid reacts rapidly with the tocopherol radical by reducing the ascorbate radical (semidehydroascorbate) to ascorbate by NADH-dependent semidehydroascorbate reductase [6,25]:

**Ascorbic acid +**^•^**Tocopherol → Ascorbate + Tocopherol Ascorbate + NADH → Ascorbate +**^•^**NAD** (1)

Although the main function of ascorbic acid is antioxidant, under certain circumstances, it can also have a pro-oxidant effect, in particular by maintaining the transition metal ions, Fe^3+^ and Cu^2+^ in their reduced forms. These metal ions react then with H_2_O_2_ to form the highly reactive ^·^OH in the Fenton reaction [25,191]. But there is currently no clear evidence that these reactions are of significance *in vivo* [25,191]:

**Fe^3+^ + Ascorbic acid → Fe^2+^ +**^•^**Ascorbate Fe^2+^ + H_2_O_2_ →**^•^**OH + OH^−^ + Fe^3+^** (2)

#### 3.3.2. Vitamin E

The term “vitamin E” does not refer to a single molecule but rather is frequently used to designate a family of chemically related compounds, namely tocopherols and tocotrienols, which share a common structure with a chromanol ring and isoprenic side chain [188]. α, β, γ, and δ Tocopherols were identified and quantified in edible mushrooms. However till now, tocotrienols were not detected in any of the available studies [6].

Biologically the most active form of vitamin E is α tocopherol whose main role is to protect cell membranes from lipid peroxidation (LPO) [25,188]. α Tocopherol terminates the activity of LPO by scavenging ^·^LOO, but during this reaction is itself converted into a reactive radical [6,25]:

**LH + Oxidant initiator →**^•^**L**^•^**L + O_2_** → ^•^**LOO**^•^**LOO + Tocopherol → LOOH +**^•^**Tocopherol** (3)

α-Tocopherol can also act as a prooxidant and reduces Fe^3+^ or Cu^2+^ [193]. The ability of α tocopherol to act as a pro- or antioxidant depends mainly on its amount which is available for scavenging of ROS [193]. Epidemiological studies indicate that diets rich in fruits and vegetables lower GI cancer rates, but supplementation of exogenous vitamin E and other antioxidants (β-carotene, vitamins A, C and Se) have not been shown to prevent GI cancers [194]. In some cases, vitamin E increases cancer risks [195]. Likewise, supplementing vitamin E has been shown to significantly increase lung cancer progression and reduce survival rate in mouse models [196]. As the expression of endogenous antioxidant genes is reduced by the introduction of antioxidant supplements, the tumor transcriptome profile is altered with a downregulation of *p53* (tumor suppressor protein gene) expression level accordingly [197]. These changes provide an explanation on why antioxidants may promote cancer development [195].

The amounts of tocopherols detected in edible mushrooms were much lower that those measured in some groceries which are usually recommended as a good source of vitamin E [6,139,147,192]. Compared with foods high in vitamin E content, e.g., almonds (26.2 mg/100 g), roasted sunflower seeds (36.3 mg/100 g), avocados (2.1 mg/100 g), tofu (5.3 mg/100 g), shrimp (2.2 mg/100 g) *etc*., the tocopherols content in edible mushrooms is measured to be between 0.02 and 200 μg/100 g DW [139,147,192,198].

#### 3.3.3. Vitamin A Including Carotenoids

Carotenoids are natural pigments. There are several dozen carotenoids in foods. The predominant carotenoids in the diet and human body are represented by β-carotene, α-carotene, lycopene, lutein and β-cryptoxanthin, of which β-carotene, α-carotene, and β-cryptoxanthin are able to function as provitamin A and play important roles as vitamin A dietary sources [199]. Recent interest in carotenoids has focused on the role of lycopene in human health. It does not have pro-vitamin A properties. Because of the unsaturated nature of lycopene it is considered to be a potent antioxidant and a ^1^O_2_ quencher. Lycopene was shown to cross the blood brain barrier and be present in the central nervous system in low concentrations. Significant reduction in the levels of lycopene was reported in Parkinson’s disease and vascular dementia patients [199,200].

Particularly, β-carotene and lutein were found in several mushroom species [6,16,201]. Carotenoids found in the pink-red *Cantharellus cinnabarinus* and the orange *Cantharellus friesii* are composed almost entirely of canthaxanthin, a pigment also found in the salmon [201]. It might explain the use of chanterelles by Chinese herbalists in treating night blindness [201]. Canthaxanthin is reported to protect human tissues from oxidative damage and is sold as an antioxidant [201].

Carotenoids can react with ROS and become radicals themselves. They function as a chain-breaking antioxidant in a lipid environment, especially under low oxygen partial pressure. The extensive systems of double bonds make carotenoids susceptible to attack ^•^LOO, resulting in the formation of inactive products [202,203].

Carotenoids reactivity depends on the length of conjugated double bonds chain and the characteristics of the end groups [202]. Carotenoid radicals are stable by virtue of the delocalization of the unpaired electron over the conjugated polyene chain of the molecules. This delocalization also allows additional reactions which occur at many sites on the radical. The carotenoid radicals are very short-lived species [202].

The antioxidant properties of carotenoids can be reversed to pro-oxidant behavior depending on oxygen pressure or carotenoid concentration [14,25,203]. It is established that β-carotene loses its antioxidant activity when the normal ambient oxygen pressure increases, and has an autocatalytic pro-oxidant effect which increases with its concentration [7,203]. Studies of food supplementation with large doses of β-carotene in smokers have shown an increase in cancer risk, possibly because β-carotene under intense oxidative stress (e.g., induced by heavy smoking) gives breakdown products that reduce plasma vitamin A and worsen the lung cell proliferation induced by smoke [149,203]. Frequent use of vitamin C and vitamin E in the period after breast cancer diagnosis was associated with a decreased likelihood of recurrence, whereas frequent use of combination carotenoids was associated with increased mortality [204].

#### 3.3.4. Vitamin D

Vitamin D refers to a group of fat-soluble secosteroids responsible for enhancing intestinal absorption of calcium (Ca), Fe, magnesium (Mg), P and Zn [205,206,207]. The importance of vitamin D in bone (Ca^2+^ ion homeostasis) is well established, and vitamin D has been the subject of increased attention in recent years for its role in muscle function, immunology, heart and cardiovascular disease, cancer, and insulin secretion [208].

In humans, the most important compounds in this group are vitamin D_3,_ also known as cholecalciferol and vitamin D_2_, or ergocalciferol. Both cholecalciferol and ergocalciferol are inactive themselves, but are metabolized in the liver to 25-hydroxyvitamin D and further in the kidney to the biologically active form, 1,2,5-dihydroxyvitamin D. The serum concentration of 25-hydroxyvitamin D is considered to be a good indicator of vitamin D status in humans [209].

Vitamin D_3_ originates from animal sources, and vitamin D_2_ is derived predominantly from mushrooms and yeast [208]. Vitamin D_2_ contents in mushrooms, cultivated or harvested wild, is available but varies significantly with and within different species and by developmental stage [210,211]. Differences of climate, habitat, and degree of latitude may cause variation in ergocalciferol contents of mushrooms [210]. For example, content of only 0–3.75 μg/100 g of fresh weight has been found in *A. bisporus* [210,212,213,214] and 0.04 [215] to 21.8–109.6 μg/100 g DW [216] in *L. edodes*.

The vitamin D_2_ content of mushrooms can be increased dramatically by UV irradiation, whereby it is formed from ergosterol that is present in large amounts [208,217,218,219,220]. Outila *et al.* [209] showed for the first time that ergocalciferol was well absorbed in humans from lyophilized and homogenized wild edible mushrooms.

Vitamin D_3_ and D_2_, its active metabolite 1,25-dihydroxycholecalciferol and also 7-dehydrocholesterol (pro-Vitamin D_3_) are a membrane antioxidants and inhibit iron-dependent liposomal lipid peroxidation [207]. The structural basis for the antioxidant ability of these vitamin D compounds is considered in terms of their molecular relationship to cholesterol and ergosterol [207].

Biologically active, 1,25-dihydroxyvitamin D, binds to the vitamin D receptor on cells and plays a role not only in Ca^2+^ uptake but also in differentiation [50,208,211]. Furthermore, by decreasing membrane fluidity by the membrane interaction that is thought to lead to the observed inhibition of iron-dependent liposomal lipid peroxidation [207], Vitamin D could help inhibit the growth of cancer cells (especially metastatic cells), which often have increased membrane fluidity compared to normal cells [221].

Van Griensven and Verhoeven reported [50] that crude polysaccharides extract of *Phellinus linteus* increased the mitochondrial membrane potential (MMP) and causes apoptotic death of THP-1 monocytes. They concluded that there are two major factors determining differentiation and/or death of polysaccharide extract treated THP-1 cells, *i.e*., a death receptor (TRAIL-like) binding activity on the one hand, and oxidative stress and Ca^2+^ homeostasis on the other. It was confirmed that *P. linteus* polysaccharides extract increased mitochondrial Ca^2+^ concentration, resulting in breakdown of the outer mitochondrial membrane and induction of apoptosis [222]. Apoptosis through the mitochondrial pathway merely seems to be caused by disturbance of the mitochondrial Ca^2+^ homeostasis [223]. Changes in the MMP and Ca^2+^ homeostasis play a prominent role in the pathogenesis of age related loss of neuronal function, such as that occurring in Alzheimer’s disease [224] and Parkinson's disease [225]. Hyperpolarizing compounds e.g., vitamine D_2_, in *P. linteus* might restore Ca^2+^ homeostasis and thereby prevent the loss of neuronal function [50].

#### 3.3.5. Ergothioneine

Ergothioneine (ET) is an unusual sulfur-containing derivative of the amino acid, histidine, which is obtained exclusively through the diet. Recently, a highly specific transporter for ET (ETT) was identified (integral membrane protein, OCTN1) in mammalian tissues, which explains abundant tissue levels of ET and implies its physiological role [226,227]. Cells lacking ETT are more susceptible to oxidative stress, resulting in increased mitochondrial DNA damage, protein oxidation and lipid peroxidation. ET is concentrated in mitochondria, suggesting a specific role in protecting mitochondrial components, such as DNA, from oxidative damage associated with mitochondrial generation of ^•^O_2_^−^. Because of its dietary origin and the toxicity associated with its depletion, ET may represent a new vitamin whose physiological roles include antioxidant protection [226,227].

Mushrooms are a primary source of ET containing from 400 to 2500 mg/g DW [17,143]. Weigand-Heller *et al.* [135] reported that ET from *A. bisporus* is bioavailable as assessed by red blood cell uptake postprandially. Consumption is associated with an attenuated postprandial triglyceride (TG) response.

By analyzing the fruiting bodies and mycelia of 29 edible and medicinal mushrooms species Chen *et al*. [17] confirmed presence of ET in all samples. The highest amount was observed among fruiting bodies of edible species, *P. citrinopileatus*, *P. ostreatus* (Korea), *P. ostreatus* (Taiwan) and *P. salmoneostramineus* (2850.7, 1829.4, 1458.4 and 1245.0 mg/kg, respectively) whereas among mycelia, *P. eryngii* contained the highest amount (1514.6 mg/kg). Dubost *et al.* [143] also found the ET content of *P. ostreatus* to be the highest (2590 mg/kg DW). It seems that mushrooms are an abundant source of ET [17,136,137,138,143].

### 3.4. Minerals

Mushrooms are generally capable of accumulating trace elements and then become their source in the food chain [228,229]. Trace elements Zn, Cu, Mn and Fe are cofactors of enzymes with antioxidant functions and are designated as antioxidant micronutrients [25]. Se is a major antioxidant in the form of selenoproteins that reduce the cytotoxic effects of ROS. GPx and selenoprotein-P are dominant Se compounds secreted in the blood [229]. In mushrooms, several Se compounds have been identified including selenomethionine, selenocysteine, Se-methylselenocysteine, selenite and seleno-polysaccharides [230,231,232].

Factors influencing the bioaccumulation of trace elements in mushrooms and the biological importance of the accumulation process itself are poorly understood. However, the following fundamentals have been recognized: natural factors (bedrock geochemistry), metalliferous areas and environmental pollution, fungal environment, bioaccumulation of trace elements in fruit bodies is highly specific: some macrofungi are able to accumulate elements much more effectively than others, the accumulation process can be highly element specific- macrofungi may even discriminate elements with similar properties and chemical behavior (homologues) [229,233]. Moreover, wild-grown edible mushrooms can accumulate minerals essential to humans in a much greater extent than cultivated edible mushroom [229].

However, it should also be kept in mind that these essential metals can also produce toxic effects when their intake is excessively elevated [228,229]. For example, *in vitro* and *in vivo* experiments have indicated that Se prevents carcinogenesis, but a high concentration of Se can be cytotoxic [234]. Se conjugates with two GSH to form the metabolite selenodiglutathione (GSSeSG), a potent compound that enhances ROS production, DNA damage and apoptosis [235]. Besides exogenous antioxidant treatments, the modulation of the endogenous antioxidant system can also be cytotoxic. Over expressed glutamate cysteine ligase (GCL), GCL catalytic subunit (GCLC) or GCL modifier subunit (GCLM) can lead to an imbalanced GSH/GSSG ratio. Excess GSH shifts the cells to reductive stress, which can cause mitochondrial oxidation and cytotoxicity [195,236].

Many edible mushroom species are known to accumulate high levels of heavy metals, mainly cadmium (Cd) and lead (Pb) [237,238,239,240]. Divalent metal cations belonging to the transition elements may pass into the mitochondria through membrane channels for essential group elements [241]. For example Pb^2+^ ions seem to be transported into human and animal mitochondria through the Ca^2+^ transporters [241,242]. Also, after reaching the cytoplasm, Pb^2+^ continues its destructive mimicking action by occupying the Ca^2+^ binding sites on numerous Ca-dependent proteins. Pb^2+^ has high affinity for Ca-binding sites in the proteins; a pM concentration of Pb^2+^ can replace Ca^2+^ in μM concentrations [242].

Cd^2+^ ions can replace Fe^2+^ and Cu^2+^ from a number of cytoplasmic and membrane proteins like ferritin, thereby causing rise in the concentration of Fe^2+^ and Cu^2+^ ions which may be associated with the production of oxidative stress via the Fenton reaction [243]. Another mechanism of Cd^2+^ toxicity may be carried out in the body by Zn^2+^ binding proteins. Cd^2+^ can bind up to ten times more strongly than Zn^2+^ in certain biological systems and is notoriously difficult to remove [242].

Another target for Pb^2+^ and Cd^2+^ attack could be a thiol group (-SH) containing enzymes involved in antioxidant mechanisms, *i.e*., Cd^2+^ forms Cd-Se complexes in the active centre of GPx and inhibits the enzyme activity [242]. Likewise, Cd^2+^ and Pb^2+^ inhibit complex III of the mitochondrial electronic transport chain, divert the electron flow and increase production of ROS [244] which may damage the mitochondrial membrane and trigger onset of apoptosis. In addition, changes in mitochondrial oxidative metabolism may lead to energy deficit thereby affecting the essential cellular functions. Thus, Cd and Pb are capable of eliciting of ROS, which could be the main mechanism of cellular toxicity induced by these heavy metals [241,242,245].

## 4. Conclusions

Oxidative stress plays a significant role in ageing processes and increases the risk of chronic diseases. The ability to resist or prevent oxidative stress is a key determinant of longevity. Enhancement of antioxidant defenses through dietary supplementation would seem to provide a reasonable approach to reduce the level of oxidative stress. There is a wealth of evidence to support the effectiveness of such a strategy *in vitro* [14].

Edible mushrooms have been related to significant antioxidant properties due to their bioactive compounds, such as polyphenols, polysaccharides, vitamins, carotenoids and minerals. Wild or cultivated, they are a primary source of ergothioneine which has specific role in protecting mitochondrial components from oxidative damage associated with generation of ^·^O_2_^−^ via the escape of electrons from the mitochondrial ETS. Likewise they are becoming increasingly important in our nutrition due to their high amounts of valuable proteins and low total fat levels, making them well suited for the prevention and treatment of obesity. Mushrooms are also very appreciated for their texture, flavor, and versatility in culinary activities. They can be easily incorporated into any kind of dish, improving the dietary diversity without adding many calories. Antioxidant and health benefits, observed in edible mushrooms, seem an additional reason for their use as a popular delicacy food. Besides, mushroom antioxidants are particularly of interest to the present generation because they have the potential to substantially reduce the expensive, high-tech, disease treatment approaches presently being employed in healthcare [2,148].

Nonetheless, successful implementation of mushroom antioxidants still remains insufficiently explored. To act *in vivo*, antioxidants would need to be incorporated into the tissues in the correct location and at a suitable concentration relative to the oxidizing agent and the molecule to be protected. In addition, some of mushroom ROS scavengers act in oxidation-reduction reactions that are reversible, and some can act both as antioxidants and pro-oxidants, depending on the conditions. Besides, the human population is heterogeneous regarding ROS levels which in moderate concentrations are necessary for a number of protective reactions. Intake of mushroom antioxidants could protect against cancer and other degenerative diseases in people with innate or acquired high levels of ROS. Abundant antioxidants might suppress these protective functions, particularly in people with a low innate baseline level of ROS [42,56,195,204,246].

Likewise, standardization of antioxidant dietary supplements from mushrooms is still in progress [13]. There are insufficient data to determine which antioxidant components are more effective and have a higher safety profile, purified mushroom extracts and fractions thereof, or the original crude. One of the main targets of mushroom antioxidants researches should be to postulate these standards.
